# RACK1 facilitates breast cancer progression by competitively inhibiting the binding of β-catenin to PSMD2 and enhancing the stability of β-catenin

**DOI:** 10.1038/s41419-023-06191-3

**Published:** 2023-10-17

**Authors:** Ruinan Tian, Jianfei Tian, Xiaoyan Zuo, Sixin Ren, He Zhang, Hui Liu, Zhiyong Wang, Yanfen Cui, Ruifang Niu, Fei Zhang

**Affiliations:** 1 Public Laboratory, Tianjin Medical University Cancer Institute and Hospital, National Clinical Research Center for Cancer, Tianjin, 300060 China; 2 Key Laboratory of Cancer Prevention and Therapy, Tianjin, 300060 China; 3 Tianjin’s Clinical Research Center for Cancer, Tianjin, 300060 China; 4 Key Laboratory of Breast Cancer Prevention and Therapy, Tianjin Medical University, Ministry of Education, Tianjin, 300060 China

**Keywords:** Breast cancer, Cell biology

## Abstract

The receptor for activated C kinase 1 (RACK1) is a key scaffolding protein with multifunctional and multifaceted properties. By mediating protein-protein interactions, RACK1 integrates multiple intracellular signals involved in the regulation of various physiological and pathological processes. Dysregulation of RACK1 has been implicated in the initiation and progression of many tumors. However, the exact function of RACK1 in cancer cellular processes, especially in proliferation, remains controversial. Here, we show that RACK1 is required for breast cancer cell proliferation in vitro and tumor growth in vivo. This effect of RACK1 is associated with its ability to enhance β-catenin stability and activate the canonical WNT signaling pathway in breast cancer cells. We identified PSMD2, a key component of the proteasome, as a novel binding partner for RACK1 and β-catenin. Interestingly, although there is no interaction between RACK1 and β-catenin, RACK1 binds PSMD2 competitively with β-catenin. Moreover, RACK1 prevents ubiquitinated β-catenin from binding to PSMD2, thereby protecting β-catenin from proteasomal degradation. Collectively, our findings uncover a novel mechanism by which RACK1 increases β-catenin stability and promotes breast cancer proliferation.

## Introduction

The receptor for activated C kinase 1 (RACK1) is a member of the tryptophan-aspartate repeat (WDR) protein family [1]. RACK1 was initially identified as a binding partner of activated PKC kinase [2]. Subsequent studies have shown that RACK1 is a multifunctional scaffolding protein that interacts with various receptor proteins and protein kinases, including AR, IGF1R, Src, FAK, PKM2 and FGFR [3–8]. Thus, RACK1 is involved in a variety of cellular signaling pathways and regulates many physiological or pathological cellular activities [9–13]. Depending on the differences in its binding proteins, RACK1 exhibits diverse functions in different tissues and cells [13–16]. Aberrant expression of RACK1 or alterations in its signaling pathways have been associated with various diseases, but the mechanisms involved remain to be elucidated.

Dysregulation of RACK1 has been implicated in the initiation and progression of various cancers. In most cases, RACK1 has been reported to promote proliferation, radiation and chemotherapy resistance, invasion and metastasis in a variety of cancers, such as lung, liver, breast, esophageal, melanoma, glioma, myeloma and neuroblastoma [17–29]. Consistently, cancer patients with higher RACK1 expression are associated with poor prognosis [22]. Hence, RACK1 acts as a tumor promoter in these types of cancers. Nevertheless, RACK1 suppresses the proliferation and metastasis of gastric cancer cells and may also act as a tumor suppressor [30]. Moreover, there are also some conflicting reports on the role of RACK1 in tumor progression. For instance, previous studies, including ours, have shown that RACK1 is required for breast cancer cell invasion and metastasis [31]; however, a recent study reported that RACK1 has a negative regulatory effect on breast cancer metastasis [10]. In addition, there are also contradictory reports on the role of RACK1 in colon cancer, with pro- and anti-cancer effects, respectively [32, 33]. The inconsistency of these reports may be related to the different genetic background of the cells or tissues used in the above-mentioned studies. Taken together, these results suggest that the function of RACK1 in cancer progression may be diverse and dependent on tissue type and context.

The mechanism whereby RACK1 regulates cancer progression remains to be elucidated. As a signaling hub, RACK1 has been associated with the activation of several cancer-related cellular signaling pathways, including PKC [34], receptor tyrosine kinase/PI3K/AKT, ERK/MAPK, integrin/Src/FAK [22], WNT/β-catenin, STAT3, and RhoA/Rho pathways [11, 12, 34–38]. Moreover, RACK1 also regulates the stability of some key tumor-associated proteins through the ubiquitin-proteasome system (UPS). Interestingly, RACK1 may play a role in promoting or inhibiting protein stability in different cell types, respectively. For instance, RACK1 promotes proteasomal degradation of HIF-1α in HEK-293T cells [39]. Likewise, RACK1 accelerates the ubiquitination-mediated degradation of β-catenin in gastric cancer cells [30]. Conversely, RACK1 enhances the stability of HDAC1/HDAC2 by suppressing their polyubiquitination [37]. Similarly, RACK1 also maintains the stability of the transcription factors c-Jun and GCM1 [40, 41]. Thus, RACK1 modulates protein stability in a cell type-specific manner. These findings also suggest that further work is needed to improve our understanding of how RACK1 plays a role in tumor initiation and progression.

Our previous studies have shown that RACK1 is essential for the drug resistance and metastasis of breast cancer cells [20]. In this study, we sought to investigate the function and potential mechanisms of RACK1 in breast cancer cell proliferation. Here, we show that RACK1 is required for the proliferation of breast cancer cells by enhancing the stability of β-catenin and activating the canonical WNT pathway. We identified PSMD2, a key component of the proteasome [42], as a novel binding partner for RACK1 and β-catenin. Mechanistically, we elucidate that RACK1 interacts with PSMD2 and prevents ubiquitinated β-catenin from binding to PSMD2, thereby protecting β-catenin from proteasomal degradation. Collectively, our findings uncover a novel mechanism by which RACK1 enhances β-catenin stability and promotes breast cancer proliferation.

## Results

### RACK1 is required for breast cancer cell proliferation in vitro and tumor growth in vivo

**Fig. 1:**
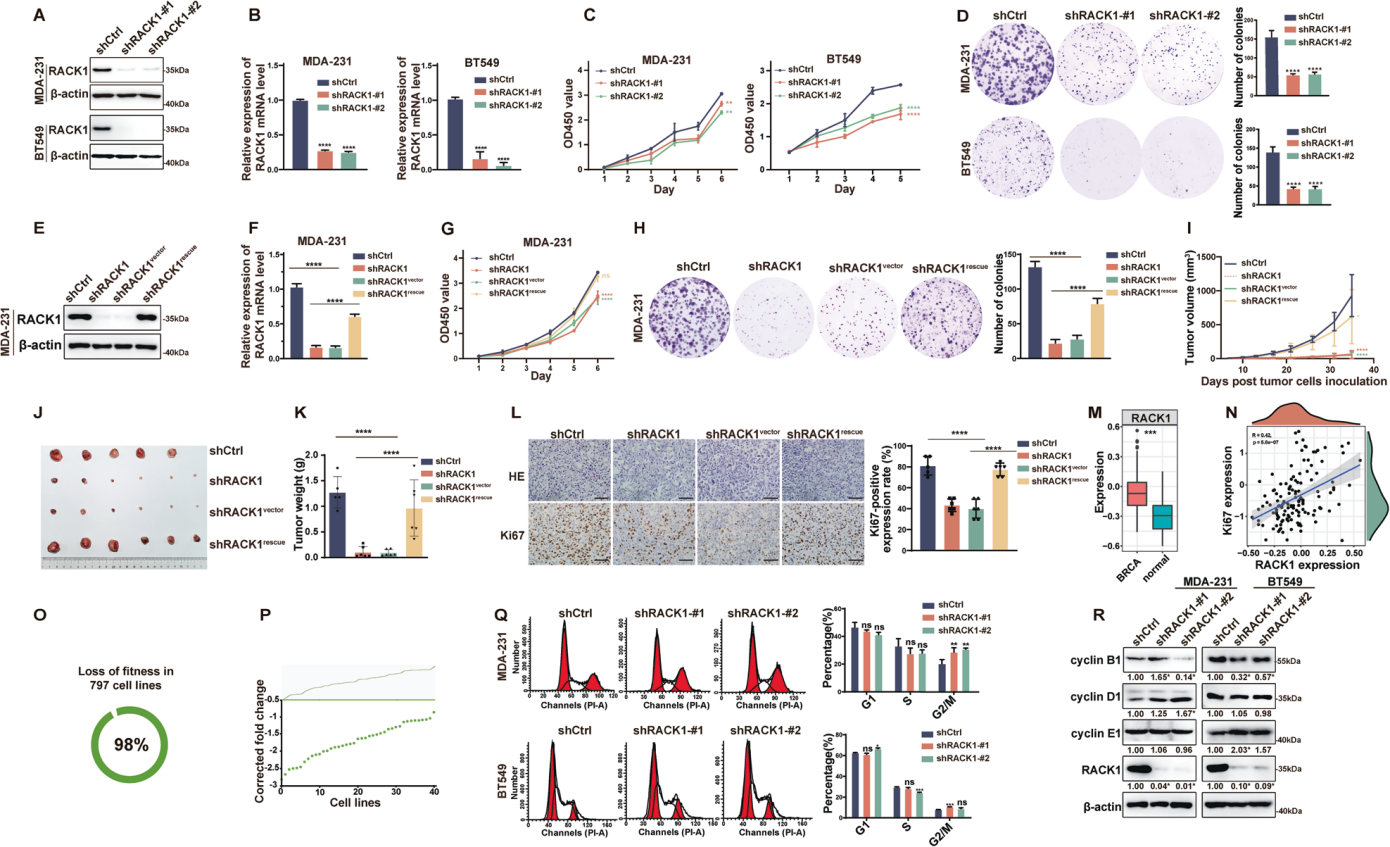
RACK1 is required for breast cancer cell proliferation in vitro and tumor growth in vivo. **A**–**B** Western blotting (**A**) and qRT-PCR (**B**) analysis of RACK1 expression in lentivirus-infected MDA-231 and BT549 cells expressing RACK1-specific shRNAs. **C** CCK8 analysis of the effect of RACK1 knockdown on the proliferative capacity of two breast cancer cell lines (two-way ANOVA test). **D** Colony formation assay showed that RACK1 knockdown decreased the proliferation rate of breast cancer cells. **E, F** Western blotting (**E**) and qRT-PCR **(F**) analysis of RACK1 expression in control, RACK1-silenced cells and RACK1-silenced cells after infection with lentivirus expressing RACK1-Flag in MDA-231 cells. **G**–**H** CCK8 **(G**) and colony formation **(H**) analysis of the proliferative capacity of control (*n* = 3), RACK1-silenced cells and cells with restored RACK1 expression in MDA-231 cells. **I** Tumor growth curves of mice inoculated with MDA-231 cells expressing different levels of RACK1 (*n* = 6). **J** Representative images of tumors from mice inoculated with the indicated MDA-231 cells. **K** The weight of tumors in the RACK1 knockdown group was significantly smaller than those in the control and rescued groups. **L** Hematoxylin and eosin (HE) and immunohistochemical (IHC) staining of Ki67 in paraffin sections of mouse tumor specimens. Scale bar, 50 μm. **M** Facet boxplot showed that the protein level expression of RACK1 was upregulated in breast cancer (BRCA) patients (*n* = 133) compared to normal tissues (*n* = 18) in the TCPA database (*P* < 0.05, LogFC > 1). **N** RACK1 was positively correlated with Ki67 expression in breast cancer tissues in the TCPA database. **O**, **P** Analysis of the Project Score database (based on genome-wide pooled CRISPR knockdown screen) revealed that RACK1 is a core fitness gene in almost all cell lines (**O**) and breast cancer cell lines (**P**). **Q** Cell cycle analysis showed that knockdown of RACK1 resulted in a significantly increased proportion of G2/M phase. **R** Western blot analysis of cyclin B1, cyclin D1 and cyclin E1 expression in control and RACK1-silenced breast cancer cells. The relative expression of the proteins was quantified by grayscale using Image J software. All data are expressed as the mean ± SD; *p < 0.05. **p < 0.01, ***p < 0.001, ****p < 0.0001 and ns *p* > 0.05 versus control, *N* = 3.

We have previously shown that RACK1 enhances the invasive and metastatic potential of breast cancer cells and modulates their drug sensitivity [20]. We then selected five breast cancer cell lines, including the triple-negative breast cancer (TNBC) cell lines MDA-MB-231 (MDA-231), BT549, and Hs578T, the luminal-type cell line T47D, and the HER2-positive cell line SK-BR-3, to investigate whether RACK1 regulates breast cancer cell proliferation. As shown in Fig. 1A, B and S1A-B, RACK1 was silenced in five breast cancer cell lines by two different lentivirally expressed shRNAs, one of which targeted the non-coding region of RACK1 for future rescue assays. Knockdown of RACK1 significantly reduced proliferation in all breast cancer cell lines as measured by CCK-8 and colony formation assays (Fig. 1C, D and S1C-D). To further confirm the function of RACK1 in cell proliferation, we infected RACK1-silenced cells with lentivirus expressing RACK1-Flag to restore its expression in MDA-231 cells (Fig. 1E, F). As expected, re-expression of RACK1 in RACK1-silenced cells significantly rescued cell proliferation in MDA-231 cells compared with control cells (Fig. 1G, H). In addition, we also upregulated RACK1 expression in MDA-231 and SK-BR-3 cells by using lentiviral infection and found that elevated RACK1 promoted cell proliferation (Fig. S1E-G). Thus, RACK1 is essential for the proliferation of breast cancer cells in vitro. To further investigate the role of RACK1 in tumor growth in vivo, we established xenograft models in BALB/c nude mice by subcutaneous injection of control and RACK1-silenced MDA-231 cells, as well as RACK1-rescued cells. As illustrated in Fig. 1I, tumor growth was very slow in the RACK1-silenced group compared with the control and RACK1-rescued groups. Thirty-five days after injection, tumors were dissected from the mice. As shown in Fig. 1J, K, the size and weight of the tumors in the RACK1 knockdown group were significantly smaller than those in the control and rescued groups. We also examined the expression of Ki67 in xenograft tumors by IHC staining. Figure 1L showed that the intensity of Ki67 was significantly decreased after RACK1 deletion. Thus, RACK1 is required for breast tumor growth in vivo. We also examined the expression of RACK1 in The Cancer Proteome Atlas (TCPA) database and found that RACK1 protein expression was higher in breast cancer tissues than in normal tissues (Fig. 1M). In addition, RACK1 was positively correlated with Ki67 expression in breast cancer tissues (Fig. 1N). Furthermore, according to data from Project Score (https://score.depmap.sanger.ac.uk/), RACK1 affected the viability of 98% of cell lines, including 41 breast cancer cell lines (Fig. 1O, P). Since the cell proliferation capacity is closely related to cell cycle progression, we subsequently examined cell cycle alterations and found a significantly increased proportion of G2/M phase in RACK1-silenced cells (Fig. 1Q). In line with these observations, knockdown of RACK1 resulted in decreased expression of cyclin B1, a key molecule in the G2/M phase transition. In contrast, the expression of cyclin D1 and E1, which regulate the G1/S phase transition, was not significantly altered (Fig. 1R). Collectively, RACK1 is required for breast cancer cell proliferation in vitro and tumor growth in vivo.

### RACK1 promotes proliferation by affecting β-catenin expression in breast cancer cells

**Fig. 2:**
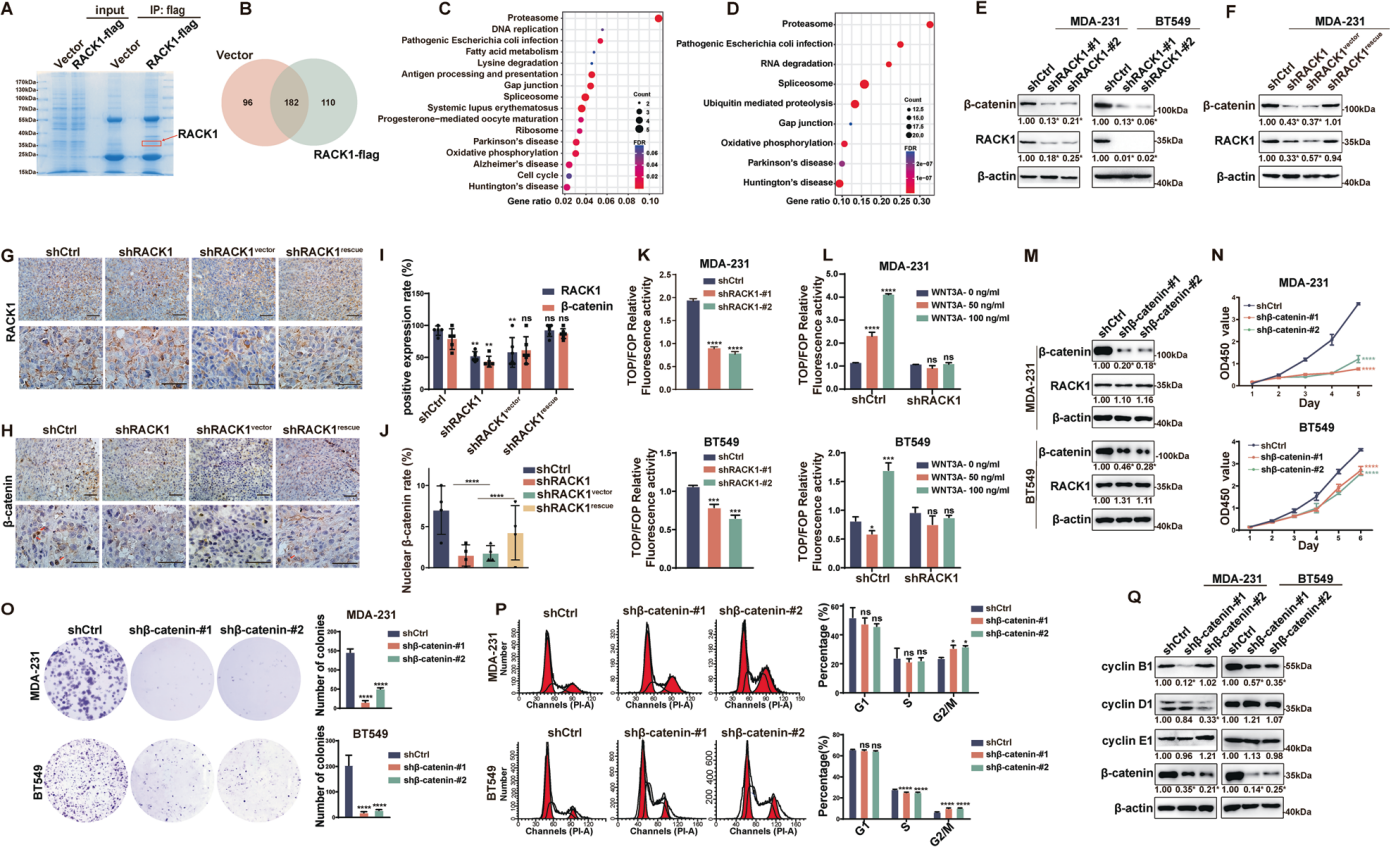
RACK1 promotes proliferation by affecting β-catenin expression in breast cancer cells. **A** Coomassie brilliant blue staining of anti-Flag antibody immunoprecipitation products from control and RACK1-flag expressing MDA-231 cells. **B** The Venn diagram of 110 proteins interacting with RACK1. **C** Bubble chart showing the KEGG enrichment analysis of RACK1-interacting proteins. **D** Bubble chart showing the KEGG enrichment analysis of RACK1 co-expression genes in the TCPA database. **E** Western blotting analysis showed that silencing of RACK1 inhibited β-catenin expression in MDA-231 and BT549 cells. **F** Western blotting analysis showed that restoration of RACK1 in RACK1-silenced cells rescued the expression of β-catenin in MDA-231 cells. **G, H** IHC staining of RACK1 and β-catenin in paraffin sections of mouse tumor specimens. Scale bar, 50 μm. **I** The positive expression rate of RACK1 and β-catenin in mouse tumor specimens. **J** The nuclear expression rate of β-catenin in mouse tumor specimens. **K, L** TOPflash assays showed that basal (**K**) and WNT3A-induced (**L**) β-catenin-dependent TCF/LEF transcriptional activity was significantly decreased in RACK1-silenced cells compared with control cells. **M** Western blotting analysis showed that silencing of β-catenin did not affect RACK1 expression in breast cancer cells. **N–O** Knockdown of β-catenin inhibited the proliferative capacity of breast cancer cells as measured by CCK-8 (**N**) and colony formation assay (**O**). **P** Cell cycle analysis of control and β-catenin-silenced breast cancer cells. **Q** Western blotting analysis of the expression of cyclin B1, cyclin D1, and cyclin E1 in control and β-catenin-silenced cells. The relative expression of the proteins was quantified by grayscale using Image J software. All data are expressed as the mean ± SD; *p < 0.05. **p < 0.01, ***p < 0.001, ****p < 0.0001 and ns *p* > 0.05 versus control, *N* = 3.

To determine how RACK1 regulates the proliferative capacity of breast cancer cells, we performed a co-immunoprecipitation (IP) assay using anti-Flag antibodies in previously obtained RACK1-flag-expressing MDA-231 cells and analyzed the IP product by mass spectrometry (Fig. 2A). A total of 110 proteins specifically interacting with RACK1 were identified (Fig. 2B). KEGG enrichment analysis showed that the proteasome pathway was significantly enriched in the RACK1 interactome (Fig. 2C). Furthermore, according to the breast cancer data in TCPA, the RACK1 co-expression gene set was also enriched in the proteasome pathway (Fig. 2D). These results suggest that the function of RACK1 may be closely related to the proteasome. It is well known that the stability of β-catenin, a classical proliferation-related molecule of WNT signaling, is regulated by the proteasome pathway. To investigate whether RACK1 has a functional regulatory effect on β-catenin, we examined the protein levels of β-catenin in RACK1-silenced cells. As shown in Fig. 2E and S1A, the knockdown of RACK1 significantly decreased the protein level of β-catenin in five breast cancer cell lines. In addition, restoration of RACK1 in RACK1-silenced cells also rescued β-catenin expression in MDA-231 cells (Fig. 2F). Moreover, in xenograft tissue specimens, the expression level of β-catenin was also significantly lower in the RACK1-silenced group than in the control group (Fig. 2G-I), and the nuclear localization of β-catenin was also lower in the RACK1-silenced group (Fig. 2J). Meanwhile, β-catenin-dependent TCF/LEF transcriptional activity was significantly decreased in RACK1-silenced cells compared with control cells as measured by TOPflash assay (Fig. 2K). Similarly, WNT3A-induced activation of the TCF reporter was also significantly reduced in RACK1-silenced cells compared with control cells (Fig. 2L). In contrast, RACK1 upregulation increased β-catenin-dependent TCF/LEF transcriptional activity and WNT3A-induced activation of the TCF reporter (Fig. S2A-B). These data indicate that RACK1 regulates β-catenin expression and activation of the canonical WNT pathway in breast cancer cells. To determine whether the effect of RACK1 on proliferation is mediated by β-catenin, we knocked down β-catenin expression and found that β-catenin silencing did not affect RACK1 protein levels (Fig. 2M and S2C). However, the proliferative capacity of β-catenin-silenced cells was significantly reduced compared to control cells (Fig. 2N, O and S2D-E). Thus, these results suggest that RACK1 acts upstream of β-catenin. We then examined changes in cell cycle and related proteins and found that β-catenin-silenced cells showed an apparent increased proportion of G2/M phase and reduced expression of cyclin B1, similar to RACK1 knockdown cells (Fig. 2P, Q). Taken together, these results suggest that RACK1 promotes breast cancer cell proliferation by affecting β-catenin protein levels and promoting cell cycle progression.

### RACK1 regulates β-catenin stability through the ubiquitin-proteasome system

**Fig. 3:**
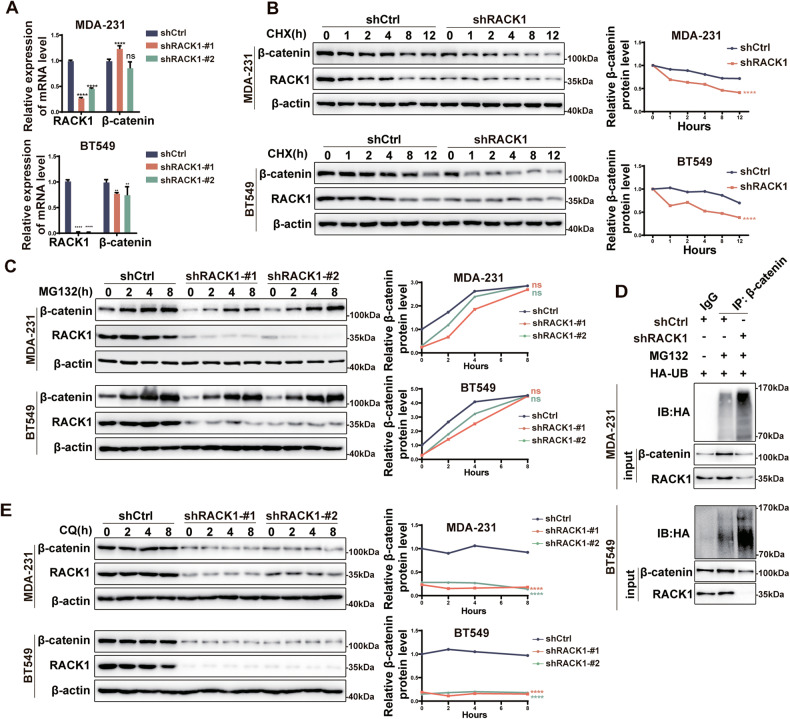
RACK1 regulates β-catenin stability through the ubiquitin-proteasome system. **A** qRT-PCR analysis of the β-catenin mRNA expression in control and RACK1-silenced breast cancer cells. **B** Western blotting analysis of β-catenin expression in control and RACK1-silenced breast cancer cells treated with cycloheximide (CHX). Gray scale analysis was performed using Image J software and the grayscale values of each band were normalized to the mean value of each group at the 0 hour time point. **C** Western blotting analysis of β-catenin expression in control and RACK1-silenced breast cancer cells treated with 10 μM of MG132. The relative expression values of the proteins were normalized to those of the control group at the 0 hour time point. **D** Knockdown of RACK1 resulted in increased levels of ubiquitinated β-catenin in two breast cancer cells. Control and RACK1-silenced cells were transfected with HA-ubiquitin and then treated with or without the proteasome inhibitor MG132. Afterwards, cell lysates were immunoprecipitated with anti-β-catenin antibodies and analyzed by Western blotting using anti-HA-ubiquitin antibodies. **E** Western blotting analysis of β-catenin expression in control and RACK1-silenced breast cancer cells treated with 50 μM of chloroquine (CQ). The relative expression values of the proteins were normalized to those of the control at the 0 hour time point. All data are expressed as the mean ± SD; ****p < 0.0001 and ns *p* > 0.05 versus control, *N* = 3.

Next, we determined whether RACK1 silencing affects β-catenin mRNA expression in two breast cancer cell lines. As shown in Fig. 3A and S1B, β-catenin mRNA levels in MDA-231, T47D, SK-BR-3, and Hs578T cells did not change significantly after RACK1 silencing, whereas β-catenin mRNA levels in BT549 cells decreased by less than 20%, suggesting that RACK1 may regulate β-catenin at the post-transcriptional level. To test this possibility, control and RACK1-silenced cells were treated with cycloheximide (CHX) to inhibit de novo protein synthesis and the half-life of β-catenin was determined. We observed a significantly faster degradation of β-catenin in RACK1-silenced cells compared with control cells (Fig. 3B and S3A). In contrast, in SK-BR-3 and MDA-231 cells, the elevation of RACK1 significantly increased β-catenin protein levels and decreased the rate of β-catenin protein degradation (Fig. S3B). These data suggest that RACK1 knockdown leads to increased β-catenin instability. To further confirm this proposal, cells were treated with a specific proteasome inhibitor, MG-132, and β-catenin levels were measured. As shown in Fig. 3C and S3C, MG-132 treatment rescued the reduction of β-catenin protein in RACK1 knockdown cells in a time-dependent manner. Moreover, inhibition of the proteasome by MG-132 also increased poly-ubiquitinated β-catenin levels in two RACK1-silenced breast cancer cells (Fig. 3D). However, treatment of RACK1-silenced cells with the autophagy-lysosome inhibitor chloroquine (CQ) did not reverse the reduction of β-catenin protein (Fig. 3E). Taken together, these results demonstrate that RACK1 regulates β-catenin protein stability through the UPS.

### PSMD2 is a novel binding partner of RACK1 and β-catenin

**Fig. 4:**
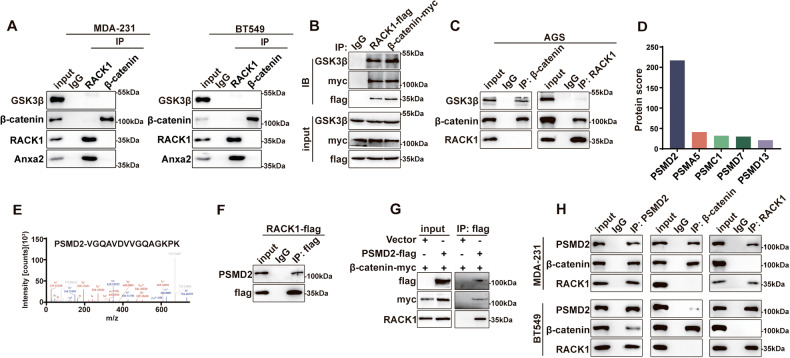
PSMD2 is a novel binding partner of RACK1 and β-catenin. **A** Co-immunoprecipitation (Co-IP) analysis of the interaction pattern among RACK1, β-catenin, GSK3β and Anxa2 in MDA-231 and BT549 cells. **B** Co-IP analysis of the interaction pattern among exogenous RACK1-flag, β-catenin-myc and GSK3β in HEK-293T cells. **C** Co-IP analysis of the interaction pattern among endogenous RACK1, β-catenin, and GSK3β in gastric AGS cells. **D** The protein score of the top 5 proteasome components identified in RACK1-interacting proteins. **E** Representative mass spectra of PSMD2. **F** Co-IP analysis of the interaction between RACK1 and PSMD2 in MDA-231 cells expressing RACK1-flag. **G** Co-IP analysis of the interaction pattern among exogenous PSMD2-flag, β-catenin-myc and RACK1 in HEK-293T cells. **H** Co-IP analysis of the interaction pattern among endogenous RACK1, β-catenin and PSMD2 in MDA-231 and BT549 cells.

β-catenin is known to be phosphorylated by GSK3β prior to ubiquitin-mediated proteasomal degradation [43]. A previous report showed that RACK1 interacts with GSK3β and promotes β-catenin degradation in gastric cancer cells [30]. To test whether this mechanism is also present in breast cancer cells, we performed a Co-IP assay in breast cancer cells using anti-RACK1 and anti-β-catenin antibodies. However, we did not identify any interaction between RACK1 and β-catenin or GSK3β in breast cancer cells (Fig. 4A). To rule out possible technical deficiencies, we performed Co-IP assays in parallel in HEK-293T and gastric cancer AGS cells. The results showed that RACK1 bound to β-catenin and GSK3β in 293 T cells (Fig. 4B). In AGS cells, we also detected β-catenin binding to GSK3β; in addition, RACK1 also bound to β-catenin (Fig. 4C). Meanwhile, as a positive control, Anxa2 was detected in the IP complex of RACK1 in MDA-231 and BT549 cells (Fig. 4A). Anxa2 has been reported to bind to RACK1 [20]. These results suggest that RACK1 does not interact with β-catenin and GSK3β in breast cancer cells. Thus, RACK1 regulates β-catenin stability through other unknown mechanisms.

Next, we further analyzed the RACK1 interactome and found that several proteasome components were highly enriched in these interacting proteins, with PSMD2 having the highest content and score (Fig. 4D, E). A Co-IP assay confirmed the interaction between FLAG-tagged RACK1 and PSMD2 in MDA-231 cells (Fig. 4F). Thus, we identified PSMD2 as a novel RACK1 binding partner. Interestingly, exogenous PSMD2 also binds to β-catenin in HEK-293T cells (Fig. 4G). We then analyzed the endogenous interaction of these proteins in breast cancer cells using Co-IP assays with anti-PSMD2, RACK1, and β-catenin antibodies. As shown in Fig. 4H, PSMD2 can precipitate RACK1 and β-catenin, and both RACK1 and β-catenin also bind to PSMD2. However, no interaction was found between RACK1 and β-catenin. Collectively, we identified PSMD2 as a novel binding partner of RACK1 and β-catenin in breast cancer cells.

### PSMD2 regulates β-catenin stability in breast cancer cells

**Fig. 5:**
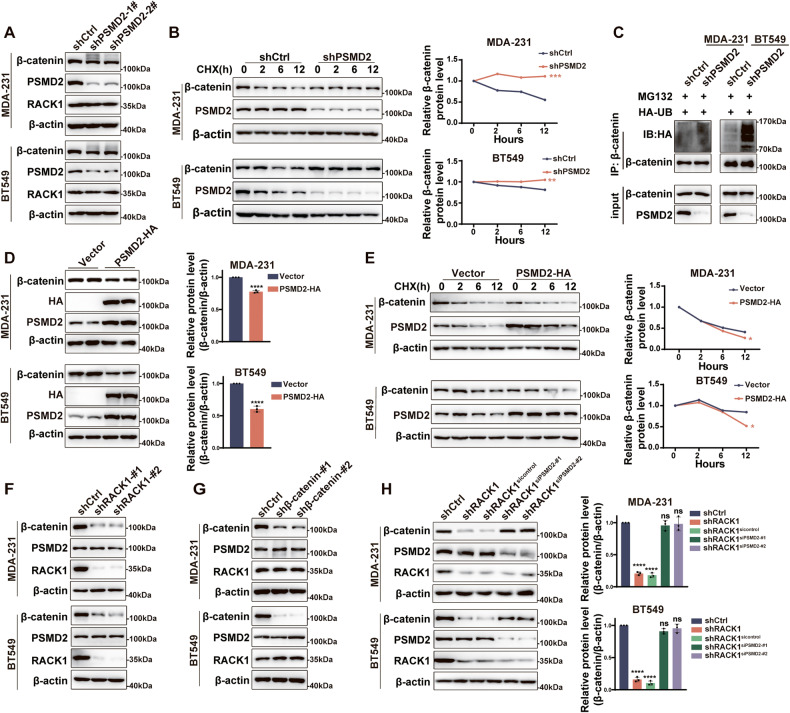
PSMD2 regulates β-catenin stability in breast cancer cells. **A** Western blotting analysis of β-catenin and RACK1 expression in PSMD2-silenced breast cancer cells. **B** Western blotting analysis of β-catenin expression in control and PSMD2-silenced breast cancer cells treated with 50 μg/mL of CHX. The relative levels of β-catenin were quantified and are shown in the right panel. **C** Knockdown of PSMD2 increased the levels of ubiquitinated β-catenin. Cell lysates were immunoprecipitated with anti-β-catenin antibodies, and then analyzed by Western blotting with anti-HA-ubiquitin antibodies. **D** Western blotting analysis of β-catenin expression in PSMD2 overexpressing MDA-231 and BT549 cells. The relative expression levels of β-catenin are shown in the right panel. **E** Western blotting analysis of β-catenin expression in control and PSMD2-overexpressing cells treated with 50 μg/mL of CHX. The relative levels of β-catenin were quantified and are shown in the right panel. **F** Western blotting analysis of PSMD2 and β-catenin expression in RACK1-silenced breast cancer cells. **G** Western blotting analysis of PSMD2 and RACK1 expression in β-catenin-silenced breast cancer cells. **H** Western blotting analysis of β-catenin expression in RACK1-silenced and RACK1 and PSMD2 dual-silenced breast cancer cells. The relative levels of β-catenin were quantified and are shown in the right panel. All data are expressed as the mean ± SD; *p < 0.05. **p < 0.01, ***p < 0.001, ****p < 0.0001 and ns *p* > 0.05 versus control, *N* = 3.

Since PSMD2 is a key subunit of the 19S proteasome, it acts as a ubiquitin receptor that recognizes substrates and delivers them to the 20S core particle for degradation [44]. While β-catenin is degraded by the proteasome, the binding between these two molecules led us to speculate that PSMD2 may regulate the stability of β-catenin. We then silenced the expression of PSMD2 in breast cancer cells. As shown in Fig. 5A, knockdown of PSMD2 did not affect the protein levels of β-catenin and RACK1 in two breast cancer cells. However, treatment of PSMD2-silenced cells with CHX substantially decreased the degradation rate of β-catenin (Fig. 5B). Interestingly, the poly-ubiquitination level of β-catenin was dramatically increased after PSMD2 knockdown (Fig. 5C). These data suggest that PSMD2 silencing affects the degradation of β-catenin but not its ubiquitination. Moreover, high expression of exogenous PSMD2 in breast cancer cells decreased the protein abundance of β-catenin (Fig. 5D) and increased the rate of β-catenin degradation (Fig. 5E). In addition, RACK1 knockdown had no apparent effect on PSMD2 expression, but decreased the level of β-catenin (Fig. 5F). In β-catenin-silenced cells, the expression of RACK1 and PSMD2 was also not altered (Fig. 5G). Interestingly, the knockdown of PSMD2 in RACK1-silenced cells rescued the reduction in β-catenin protein levels caused by RACK1 depletion (Fig. 5H). Together, PSMD2 acts upstream of β-catenin and regulates its stability in breast cancer cells.

### PSMD2 interacts with ubiquitinated β-catenin

**Fig. 6:**
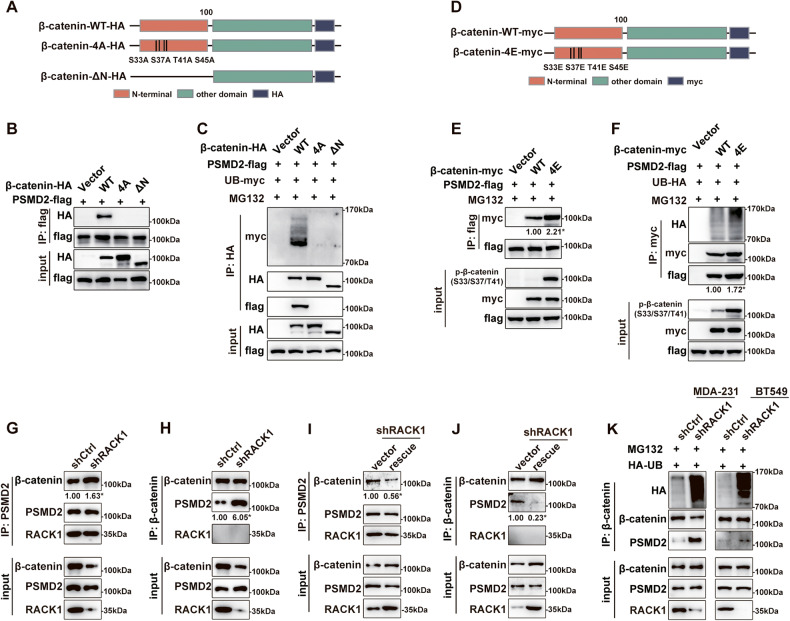
PSMD2 interacts with ubiquitinated β-catenin. **A** Diagram of full-length HA-tagged β-catenin and its two mutants, S33A/S37A/T41A/S45A (4A) β-catenin and N-terminal truncated (ΔN) β-catenin. **B** Co-IP analysis of the interaction pattern between PSMD2 and the indicated β-catenin mutants in HEK-293T cells. **C** PSMD2 interacts with ubiquitinated β-catenin. HEK-293T cells were co-transfected with PSMD2-Flag, UB-myc and β-catenin-HA or its mutants and then treated with MG132, followed by immunoprecipitation of cell lysates with anti-HA antibodies and detection with anti-HA, -myc and -Flag antibodies. **D** Diagram of full-length myc-tagged β-catenin and S33E/S37E/T41E/S45E (4E) β-catenin. **E** Co-IP analysis of the interaction pattern between PSMD2 and β-catenin-WT-myc or β-catenin-4E-myc in HEK-293T cells. **F** PSMD2 interacts with ubiquitinated β-catenin. HEK-293T cells were co-transfected with PSMD2-Flag, HA-UB and β-catenin-WT-myc or β-catenin-4E-myc and then treated with MG132, followed by immunoprecipitation of cell lysates with anti-myc antibodies and detection with anti-HA, -myc and -Flag antibodies. **G, H** Knockdown of RACK1 increased the interaction between PSMD2 and β-catenin in MDA-231 cells. Cell lysates from control or RACK1-silenced cells were immunoprecipitated with ant-PSMD2 (**G**) or anti-β-catenin (**H**) antibodies and analyzed by Western blotting with anti-PSMD2, RACK1, or β-catenin antibodies. **I, J** Restoration of RACK1 expression in RACK1-silenced cells attenuated the interaction between PSMD2 and β-catenin. Cell lysates from RACK1-silenced or RACK1-rescued cells were immunoprecipitated with ant-PSMD2 (**I**) or anti-β-catenin (**J**) antibodies and analyzed by Western blotting with anti-PSMD2, RACK1, or β-catenin antibodies in MDA-231 cells. The protein levels of the IP products were quantified by grayscale analysis and the results are shown below the bands (**E**-**J**). All data are expressed as the mean ± SD; *p < 0.05 and ns *p* > 0.05 versus control, *N* = 3. **K** Knockdown of RACK1 increased the binding ability of PSMD2 to ubiquitinated β-catenin in breast cancer cells. Control and RACK1-silenced cells were transfected with UB-HA and then treated with MG132. Afterwards, cell lysates were immunoprecipitated with anti-β-catenin antibodies and analyzed by Western blotting using anti-PSMD2, RACK1, HA or β-catenin antibodies.

To investigate the underlying mechanism by which PSMD2 regulates β-catenin stability, as shown in Fig. 6A, we constructed full-length HA-tagged β-catenin (abbreviated as WT) and its two mutants, namely phospho-deficient β-catenin-S33A/S37A/T41A/S45A (where S33/S37/T41/S45 were mutated to A, abbreviated as 4A) and N-terminal truncated β-catenin-ΔN (100 amino acids deleted at the N-terminus). These constructs were then co-transfected with Flag-tagged PSMD2 into HEK-293T cells and Co-IP was performed with anti-Flag antibodies. As shown in Fig. 6B, C, PSMD2 only interacted with WT β-catenin and did not bind to either mutant. Because these two mutants cannot be phosphorylated or ubiquitinated [45], these data suggest that PSMD2 may be more likely to interact with phosphorylated or ubiquitinated β-catenin. Next, As shown in Fig. 6D, full-length myc-tagged β-catenin (abbreviated as WT) and phospho-mimetic β-catenin-S33E/S37E/T41E/S45E (S33/S37/T41/S45 mutated to E, abbreviated as 4E) were constructed and co-transfected with Flag-tagged PSMD2 into HEK-293T cells, followed by Co-IP with anti-Flag antibodies. As shown in Fig. 6E, PSMD2 bound more to β-catenin-4E than to β-catenin-WT. Moreover, the ubiquitination of β-catenin-4E was significantly enhanced, and the binding ability of β-catenin-4E to PSMD2 was higher than that of β-catenin-WT to PSMD2 (Fig. 6F). In addition, immunofluorescence assay showed that β-catenin-4E co-localized more efficiently with PSMD2 than β-catenin-WT with PSMD2 (Fig. S4). Since β-catenin is known to be phosphorylated prior to ubiquitination. Taken together, these data suggest that PSMD2 tends to interact with phosphorylated and ubiquitinated β-catenin. Since RACK1 silencing increased the ubiquitination of β-catenin (Fig. 3D), we next examined the ability of PSMD2 to bind to β-catenin in RACK1-silenced cells by a Co-IP assay using anti-PSMD2 antibodies. As shown in Fig. 6G, anti-PSMD2 antibodies co-precipitated more β-catenin in RACK1-silenced cells compared to control cells in MDA-231 cells. Consistently, a reciprocal Co-IP assay also showed that anti-β-catenin antibodies co-precipitated more PSMD2 in RACK1-silenced cells than in control cells (Fig. 6H) and that restoration of RACK1 expression in RACK1-silenced cells reversed this phenomenon in MDA-231 cells (Fig. 6I, J). In addition, we treated RACK1-silenced cells with MG132 and found that the binding ability of PSMD2 to poly-ubiquitinated β-catenin was significantly enhanced in both breast cancer cells (Fig. 6K). Together, these results suggest that RACK1 knockdown facilitates the binding between PSMD2 and ubiquitinated β-catenin.

### RACK1 competes with β-catenin for binding to the Armadillo-like helical domain of PSMD2

**Fig. 7:**
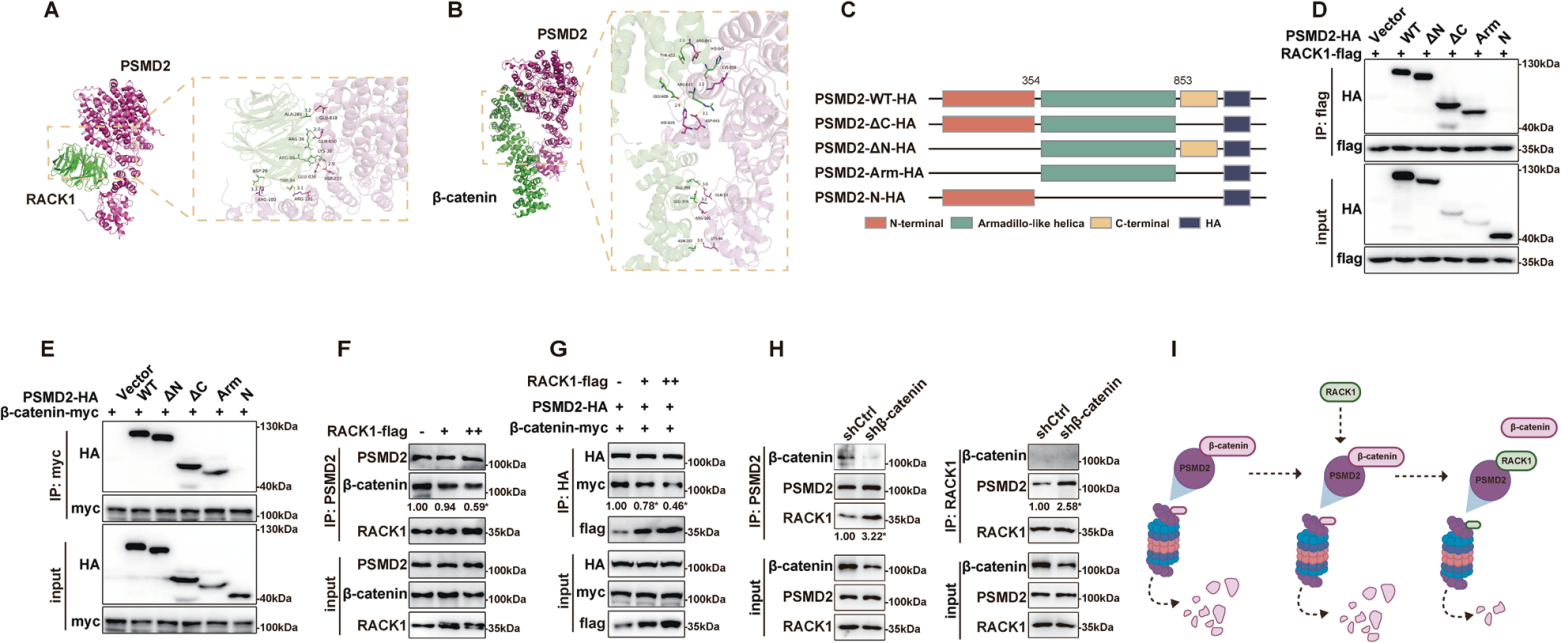
RACK1 competes with β-catenin for binding to the Armadillo-like helical domain of PSMD2. **A**, **B** Molecular docking analysis of the binding pattern of RACK1 to PSMD2 (A) or β-catenin to PSMD2 (B). The protein backbone is rendered in tube and colored in green (β-catenin or RACK1) and red (PSMD2). The target proteins RACK1 (PDB ID: 4AOW), β-catenin (PDB ID: 7AR4) and PSMD2 (PDB ID:6MSD) were obtained from the RCSB database (https://www.rcsb.org/). **C** Schematic representation of HA-tagged full-length PSMD2 and its fragments containing different domains. **D** Co-IP analysis of the interaction pattern between RACK1 and the indicated PSMD2 mutants. HEK-293T cells were cotransfected with RACK-Flag and PSMD-HA or its mutants, then cell lysates were immunoprecipitated with anti-Flag antibodies and analyzed by Western blotting using anti-HA or Flag antibodies. **E** Co-IP analysis of the interaction pattern between β-catenin and the indicated PSMD2 mutants. HEK-293T cells were cotransfected with β-catenin-myc and PSMD-HA or its mutants, then cell lysates were immunoprecipitated with anti-myc antibodies and analyzed by Western blotting using anti-HA or myc antibodies. **F** Co-IP analysis of the interaction pattern between endogenous β-catenin and PSMD2 in the presence of elevated RACK1 expression. HEK-293T cells were transfected with increasing amounts of RACK1 plasmids, and the cell lysates were subjected to Co-IP with anti-PSMD2 antibodies and then analyzed with anti-PSMD2, RACK1 and β-catenin antibodies. **G** Co-IP analysis of the interaction pattern between exogenous β-catenin and PSMD2 in the presence of elevated RACK1 expression. **H** Knockdown of β-catenin increased the interaction between PSMD2 and RACK1 in MDA-231 cells. The protein levels of the IP products were quantified by gray scale analysis and the results are shown below the bands (**F**-**H**). All data are expressed as the mean ± SD; *p < 0.05 and ns *p* > 0.05 versus control, *N* = 3. **I** Diagram showing that RACK1 competes with β-catenin to bind PSMD2.

To identify the specific domain of the PSMD2 protein involved in the interaction with RACK1 and β-catenin, we performed molecular docking analysis and found that the Armadillo-like helical domain (355 to 853 amino acids) may play a key role. Molecular docking revealed that several sites of PSMD2 (GLU-618, GLU-836, ARG-100, ARG-181, GLN-650, ASP-222) might be involved in the interaction with RACK1 (Fig. 7A), and other sites of PSMD2 (ASP-645, LYS-858, LYS-66, GLN-53, ARG-845, HIS-649, ARG-100) might interact with β-catenin (Fig. 7B). Although the predicted binding sites of RACK1 and β-catenin to PSMD2 are not identical, they are relatively close and the binding of RACK1 to PSMD2 has the potential to spatially inhibit its interaction with β-catenin. To further explore the details of the interactions between RACK1, β-catenin and PSMD2, we then determined the interacting domains between these proteins. We constructed a series of vectors expressing HA-tagged full-length PSMD2 and fragments containing different domains (Fig. 7C). These vectors were then co-transfected with flag-tagged RACK1 or myc-tagged β-catenin into HEK-293T cells and Co-IP assays were performed. As shown in Fig. 7D, only the PSMD2 fragment containing the Armadillo-like helical domain interacted with RACK1 in HEK-293T cells. Interestingly, β-catenin also precipitated full-length PSMD2 and its fragments containing the Armadillo-like domain in HEK-293T cells (Fig. 7E). Thus, both RACK1 and β-catenin bind to the Armadillo-like helical domain of PSMD2. These results also suggest that RACK1 has the potential to bind PSMD2 in competition with β-catenin. Consistently, elevated RACK1 expression attenuated the interaction of PSMD2 with β-catenin in a dose-dependent manner in HEK-293T cells (Fig. 7F, G). Furthermore, silencing of β-catenin increased the interaction between PSMD2 and RACK1 in MDA-231 cells (Fig. 7H). Collectively, RACK1 competes with β-catenin for binding to the Armadillo-like helical domain of PSMD2 in breast cancer cells (Fig. 7I).

## Discussion

**Fig. 8:**
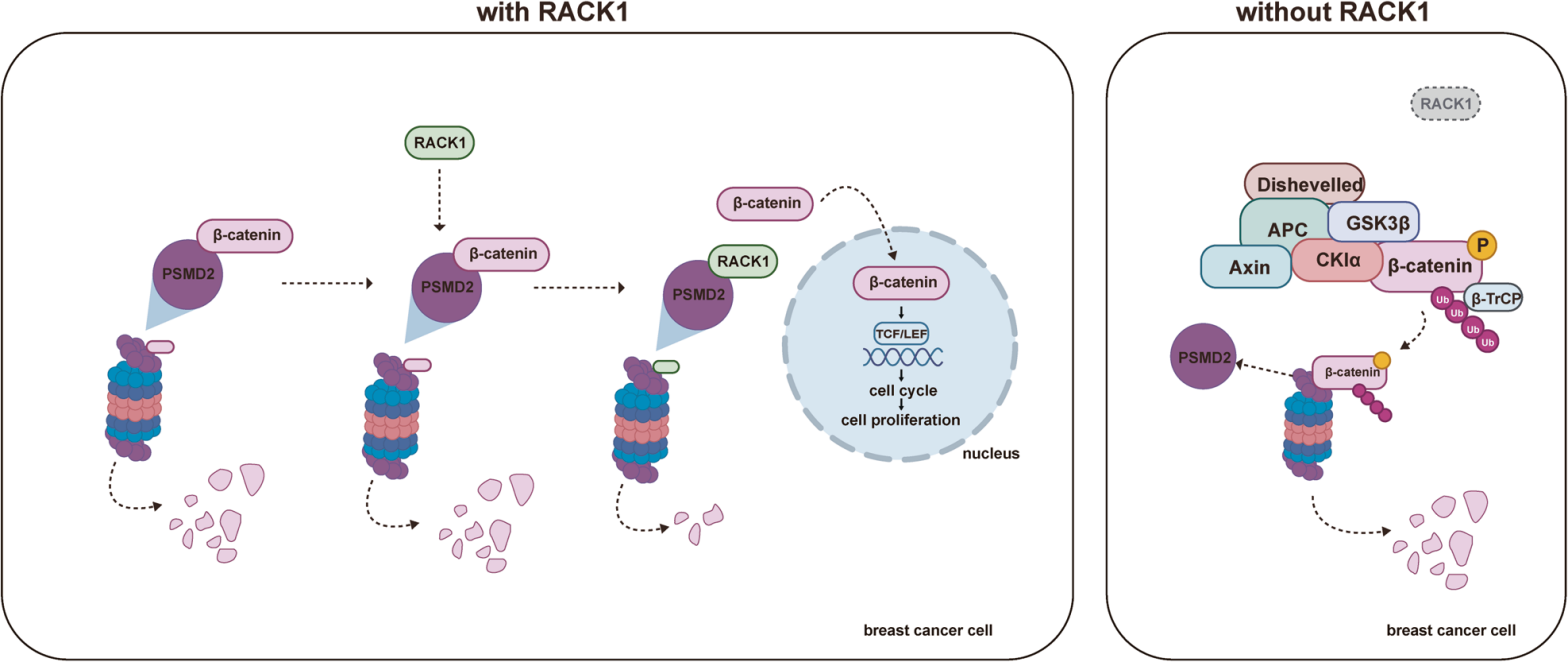
Diagram of the mechanism. RACK1 competitively inhibits β-catenin binding to PSMD2, enhances β-catenin stability, regulates cell cycle progression and facilitates breast cancer progression. PSMD2 is a key component of the proteasome that binds to β-catenin and regulates its degradation through the UPS (left). RACK1 deficiency results in increased ubiquitin-proteasome degradation of β-catenin (right).

The present study supports the tumor-promoting role of RACK1 in breast cancer. Our in vitro and in vivo evidence suggests that RACK1 is required for breast cancer cell proliferation by regulating cell cycle progression. We uncovered that RACK1 promotes the stability of β-catenin, thereby facilitating the activation of the canonical WNT pathway in breast cancer cells. We identified PSMD2 as a novel binding partner for RACK1 and β-catenin. Mechanistic investigation demonstrated that RACK1 interacts with PSMD2 and prevents ubiquitinated β-catenin from binding to PSMD2, thereby protecting β-catenin from UPS-mediated degradation. Collectively, our findings suggest that RACK1 facilitates breast cancer progression by competitively inhibiting β-catenin binding to PSMD2, increasing β-catenin stability, and promoting WNT pathway activation (Fig. 8).

Although RACK1 has been implicated in the initiation and progression of many tumors, the exact function of RACK1 in cancer cellular processes, particularly in proliferation, remains controversial. Initial studies showed that elevated RACK1 inhibited the proliferation of transformed NIH-3T3 cells [46]. Subsequently, RACK1 was reported to suppress the growth and promote apoptosis of some cancer cells, including colon, gastric and cervical squamous cancers [30, 33, 47–51]. In contrast, other studies have shown that RACK1 promotes cell proliferation in some malignancies, including prostate, breast, lung, cervical, liver, melanoma, and neuroblastoma [3, 17, 18, 21–23, 25, 50]. These inconsistent results suggest that the role of RACK1 in regulating proliferation may be cell-type dependent. Herein, RACK1 silencing inhibited breast cancer proliferation in vitro and tumor growth in vivo, whereas restoration of RACK1 expression in RACK1-deficient cells rescued the proliferative activity, supporting that RACK1 is required for breast cancer cell proliferation. Moreover, RACK1 silencing induced an apparent increase in the G2/M phase ratio, similar to previous reports that RACK1 knockdown led to an increase in the G2/M phase ratio, despite the fact that RACK1 inhibited proliferation in their cell model (non-breast cancer) [33]. In addition, a recent study showed that RACK1 is essential for the proliferation of multiple myeloma cells and that RACK1 silencing also induces an increase in G2/M phase [28, 52]. Collectively, our findings suggest that RACK1 promotes breast cancer cell proliferation by regulating cell cycle progression.

β-catenin is a well-known key proliferation-related regulator downstream of the WNT pathway, and its stability is regulated by the UPS [53]. Here, we found that RACK1 silencing significantly increased the ubiquitination level of β-catenin and accelerated its degradation in breast cancer cells and vice versa, suggesting that RACK1 regulate the stability of β-catenin protein through the UPS. Likewise, RACK1 is required for the basal level and WNT3A-induced activation of the canonical WNT pathway. Thus, RACK1 promotes the WNT signaling pathway by enhancing β-catenin stability. Moreover, silencing of β-catenin inhibited breast cancer cell proliferation similarly to RACK1 knockdown, whereas β-catenin knockdown did not affect RACK1 expression, suggesting that RACK1 acts upstream of β-catenin. Notably, previous studies have shown that RACK1 is an inhibitor of β-catenin stability and WNT signaling in gastric cancer cells [30]. This contradiction may be partly due to the different genetic backgrounds of different types of tumor cells. As a scaffolding protein, the binding partners of RACK1 in gastric cancer cells may be very different from those in breast cancer cells, resulting in a potentially different or even opposite role for RACK1 in downstream signaling regulation [2]. This possibility is partly supported by a recent study, which found that in gastric cancer cells, RACK1 is a short-lived protein that can be ubiquitinated and degraded by the ubiquitin-conjugating enzyme UBE2T [36]. Similarly, the ubiquitin E3 ligase RAB40C regulates the degradation of RACK1 in colon cancer cell lines [54]. Thus, RACK1 may function as a substrate for the UPS in these types of cancer cells. Nevertheless, the RACK1-interacting proteins identified in our study are highly enriched in proteasomal and ribosomal components, with few enzymes regulating ubiquitination modifications. In addition, we found that RACK1 is associated with ribosomal components, which is consistent with previous studies showing that RACK1 is a ribosomal protein. Interestingly, several studies have shown that ribosomal RACK1 plays a role in promoting the progression of breast cancer, neuroblastoma and hepatocellular carcinoma [17, 24, 55]. However, whether ribosomal RACK1 regulates the stability of β-catenin in breast cancer needs to be further investigated in the future. Taken together, these results suggest that RACK1 promotes breast cancer proliferation by regulating β-catenin stability and WNT pathway activation.

The mechanisms by which RACK1 regulates β-catenin stability in breast cancer cells are worthy of investigation. Although a previous study has shown that in gastric cancer cells, RACK1 promotes β-catenin degradation by interacting with GSK3β and stabilizing the β-catenin destruction complex [30]. Nevertheless, after excluding technical biases as much as possible, we did not find any interaction of RACK1 with β-catenin or GSK3β in breast cancer cells. These results suggest that the regulatory mechanism of RACK1 on β-catenin in breast cancer cells is different from that in gastric cancer cells. Herein, we found that a key subunit of the 19S proteasome, PSMD2, is a novel binding partner for RACK1 and β-catenin, although there is no direct interaction between RACK1 and β-catenin. PSMD2 has been reported to recognize ubiquitinated substrates and transport them to the catalytic 20S proteasome for proteolysis [56–58]. Therefore, we speculated that the binding of PSMD2 to β-catenin may regulate the degradation of β-catenin. As expected, silencing of PSMD2 attenuated the degradation rate of β-catenin and increased its protein level, whereas overexpression of PSMD2 decreased the stability of β-catenin in breast cancer cells. Interestingly, the knockdown of RACK1 increased the binding ability between PSMD2 and β-catenin, raising the possibility that RACK1 may competitively binds PSMD2 with β-catenin or that the binding of RACK1 to PSMD2 may inhibit the interaction between PSMD2 and β-catenin. Consistent with this conjecture, the results from biochemical assays confirmed that both RACK1 and β-catenin bind to the armadillo-like helical domain of PSMD2, and that elevated RACK1 decreased β-catenin binding to PSMD2. In addition, the results of molecular docking analysis also indicated that the armadillo-like helical domain of PSMD2 may be a common binding region for RACK1 and β-catenin, albeit with some differences. These data also partially support the possibility that RACK1 may inhibit the interaction between PSMD2 and β-catenin. Collectively, our results suggest that RACK1 interacts with PSMD2 and prevents β-catenin from binding to PSMD2, thereby protecting β-catenin from UPS-mediated degradation.

In conclusion, our results suggest that RACK1 facilitates breast cancer progression by enhancing the stability of β-catenin. We identified PSMD2 as a novel binding partner of RACK1 and β-catenin, and although RACK1 did not bind to β-catenin, it inhibited the interaction of PSMD2 with β-catenin and the subsequent proteasome-mediated degradation. Thus, we reveal a novel mode of interaction between PSMD2, RACK1 and β-catenin, that may be a mechanism involved in regulating the activation of the WNT pathway, and targeting this pathway may be an effective way to treat breast cancer.

## Materials and Methods

### Cell lines and cell culture

Human breast cancer cell lines MDA-MB-231 (MDA-231), BT549 and human embryonic kidney 293T (HEK-293T) were purchased from the American Type Culture Collection (ATCC, Manassas, VA, United States). MDA-231 and BT549 cells were grown in RPMI-1640 medium (Hyclone, South Logan, UT, United States); HEK-293T cells were cultured in DMEM medium, and all media were supplemented with 10% fetal bovine serum (HyClone) under standard culture conditions (5% CO_2_ at 37 °C).

### Plasmid construction, lentivirus production, stable cell line generation and siRNAs

Two efficient lentiviral vectors were previously constructed for the expression of RACK1-specific shRNAs. One shRNA targets the coding region of RACK1 (shRACK1-#1) and the other targets its non-coding region (shRACK1-#2). The β-catenin-specific shRNA sequences were cloned into the lentiviral vector pLKO.1- puromycin of the BamH I and Age I cloning sites. The shRNA sequences are shown in Supplementary Table 1. Lentiviruses were produced from a standard three-plasmid system as described previously. The virus was then used to infect MDA-231 or BT549 cells, and RACK1 or β-catenin stable knockdown cells were generated by using 1 µg/ml of puromycin. Two PSMD2-specific siRNAs and a negative control siRNA were purchased from Genepharma (Suzhou GenePharma Co., Ltd.). Transfection was performed using Lipofectamine RNAiMAX reagent (Invitrogen) according to the manufacturer’s instructions. The lentiviral plasmid pCDH-RACK1-Flag was constructed in our previous study. Full-length HA- or MYC-tagged β-catenin, and full-length HA- or Flag-tagged PSMD2 were generated by using polymerase chain reaction (PCR) amplification, and cloned into the pcDNA3.1 vector on EcoR I by using the ClonExpress Ultra One Step Cloning Kit (Vazym, China). Truncation mutants of β-catenin and PSMD2 were created by overlapping PCR, and point mutations of β-catenin (S33A/S37A/T41A/S45A and S33E/S37E/T41E/S45E) were introduced by PCR-based site-directed mutagenesis. All vectors were verified by Sanger sequencing. The DNA oligos used for vector construction are shown in Supplementary Table 1. Plasmid transfections were performed using Lipofectamine 3000 (Thermo Fisher Scientific, CA, USA), according to the manufacturer’s instructions.

### Western blot analysis

Isolation, quantification, SDS-PAGE and transfer protocols for total cellular proteins were performed as previously described [59]. Briefly, membranes were blocked with 5% nonfat milk in tris-buffered saline (TBS) containing 0.1% Tween 20 (TBS-T) for 1 hour at room temperature. After washing with 1× TBST, the membranes were incubated overnight at 4 °C with the following primary antibodies: RACK1 (Santa, sc-17754; CST, 5432s), β-catenin (CST, 8480s), cyclin B1 (Santa, sc245), cyclin D1 (CST, 2922s), cyclin E1 (CST, 20808s), HA tag (Abcam, ab137838), PSMD2 (Abcam, ab140675), GSK3β (CST,12456s), myc tag (Origene, TA300002), flag tag (Santa, sc-166355), β-actin (Sigma, A1978), and p-β-catenin-S33/S37T41 (CST, 9561s). After further washing, the membranes were incubated with horseradish peroxidase-conjugated secondary antibodies, and the protein bands were detected by chemiluminescence using an ECL kit (BIO-RAD, USA).

### Reverse transcription and quantitative polymerase chain reaction (qRT-PCR)

The detailed method for quantitative real-time PCR (qRT-PCR) was performed as described previously [49]. Briefly, cells were lysed using TRIzol reagent (Life Technologies, Carlsbad, CA, United States), and total RNA was isolated and reverse transcribed into cDNA by HiScript II QRT SuperMix (Vazyme, China). All qRT-PCRs were performed using AceQ qPCR SYBR Green Master Mix (Vazyme, China). The relative expression of genes was normalized to β-actin and repeated three times. Primers are listed in Supplementary Table 1.

### Cell proliferation assay

Cell proliferation capacity was determined by the Cell Count Kit 8 (CCK8) assay and the colony formation assay. The CCK8 assay was carried out as previously described [60]. Briefly, the cells were seeded in triplicate into 96-well plates at a density of 700 cells/well (MDA-231) or 1200 cells/well (BT549). At the indicated time points, CCK-8 solution (10 μL) was added to the medium, and the cells were further cultured for 4 h, then the absorbance was measured at 450 nm. For colony formation assay, cells were plated at a density of 500 cells/well (MDA-231) or 1000 cells/well (BT549) in 6-well plates. After 7–10 days, the cells were washed with PBS, fixed with methanol for 10 min, and stained with 0.1% crystal violet solution for 15 min. Plates were air dried and visible colonies were counted. The experiments were repeated 3 times in triplicate.

### Nude mice xenograft models

Four- to five-week-old of female BALB/nude mice were purchased from Gempharmatech Co., Ltd (Jiangsu, China) and randomized into four groups. Approximately 5 × 10^6^ cells were injected orthotopically into the mammary fat pad. Seven days after the inoculation, the tumors became detectable. Tumor volume was measured every 3 days and the mice were sacrificed after 35 days. Tumor volume (cm^3^) was measured by using the formula: 0.5 × smallest diameter × largest diameter^2^. The tumors were then dissected, cleaned, imaged, fixed in formalin, embedded in paraffin, and serially sectioned. The expression of RACK1, β-catenin and Ki67 was detected by immunohistochemical staining according to a standard protocol as described previously. The following antibodies were used in this experiment. RACK1 (Santa, sc-17754, 1:200), β-catenin (CST, 8480s, 1:200), and Ki67 (ready-to-use, OriGene Technologies, Inc., TA800648). All experimental operations complied with the requirements of the Animal Ethics Committee of Tianjin Medical University Cancer Institute and Hospital.

### Cell cycle analysis

Cells were fixed with 75% ethanol at 4 °C overnight. Cells were then centrifuged, washed with PBS, and stained with propidium iodide (50 μg/mL) in the presence of RNase A for 30 min at 37 °C. A total of 30,000 cells were analyzed using a FACScan flow cytometer in triplicate (BD, Franklin Lakes, NJ, USA).

### Co-immunoprecipitation assay (Co-IP)

Co-IP analysis was performed as described previously [61]. In brief, cells were solubilized with lysis buffer (40 mM Tris, 150 mM NaCl, 1% Triton X-100, 50 mM NaF, 5 mM Na_3_VO_4_, 2 mM EDTA, and 1 × protease inhibitor cocktail), and the lysates were centrifuged at 12,000 × *g* for 15 min at 4 °C. The supernatant was precleared with protein A/G agarose (Millipore) and then incubated with 1 μg of antibody overnight at 4 °C. The immunocomplex was incubated with protein A/G agarose beads for 1 h at room temperature. The beads were then washed three times with cell lysis buffer. The final pellets were resuspended in 1× SDS sample buffer, and then analyzed by Western blotting.

### Mass spectrometric analysis

Tandem mass-tag (TMT)-based quantitative proteomics analysis was performed by PTM Biolabs (Hangzhou, China) as described previously [62]. In brief, denatured IP samples were separated by electrophoresis and separately cut into gel slices. The gel pieces were destained in 50 mM NH_4_HCO_3_, dehydrated with 100% acetonitrile (ACN), and then reduced with 10 mM dithiothreitol. Afterwards, the samples were dehydrated again in 100% ACN and alkylated with 55 mM iodoacetamide. After washing with 50 mM NH_4_HCO_3,_ the samples were dehydrated with ACN, and digested with trypsin overnight at 37 °C. Tryptic peptides were extracted with 50% ACN/5% formic acid (FA), dried to the completeness and resuspended in 2% ACN/0.1% FA. Then, the peptides were dissolved in 0.1% FA (solvent A), loaded directly onto a home-made reversed-phase analytical column (15-cm length, 75 μm i.d.), and eluted on an EASY-nLC 1000 UPLC system at a constant flow rate of 400 nL/min. The gradient consisted of increasing from 6% to 23% solvent B (0.1% FA, 98% ACN) in 16 minutes, increasing from 23% to 35% in 8 minutes, climbing to 80% in 3 minutes, and then remaining at 80% for the final 3 minutes. The eluted peptides were then subjected to the NSI source and analyzed by tandem mass spectrometry (MS/MS) on a Q Exactive^TM^ Plus (Thermo) coupled online to the UPLC. Finally, the resulting MS/MS data were processed using Proteome Discoverer 1.3. The tandem mass spectra were searched against the KEGG database. Raw data has been provided in the Supplemental Table 2.

### TOP/FOP dual-luciferase reporter assay

The dual-luciferase reporter assay was performed using a commercial kit (Vazyme, DL101–01, China) following the manufacturer’s instructions. In brief, cells were plated in triplicate in 6-well plates. The next day, 2.5 ng of firefly luciferase vector (TOPflash or FOPflash) and 0.25 ng of Renilla luciferase vector were co-transfected into each well of cells, and 48 hours later, cell lysates were collected and measured by luminometer, repeated three times. Raw data has been provided in the Supplemental Table 3.

### Protein-protein docking

The 3D structures of RACK1 (PDB ID: 4AOW), β-catenin (PDB ID: 7AR4), and PSMD2 (PDB ID: 6MSD) were obtained from the RCSB database (https://www.rcsb.org/). All protein structures were processed in the Molecular Operating Environment (MOE 2019.1) platform with Amber10 stand selection, including removal of water and ions, protonation, addition of missing atoms and complementation of missing groups, and protein energy minimization. Using HDOCK software, the protein was set to rigid, the docking contact site was set to full surface, the conformation generated after docking was set to 100, and the conformation with the most negative energy was selected using the scoring function. Pymol 2.1 software was used for visualization.

### Bioinformatics analysis and statistical analysis

The publicly available TCPA-BRCA database (https://www.tcpaportal.org/) was used to determine the protein expression of RACK1 and RACK1 co-expression genes. The statistical software R (version 3.4.1) and the R packages “ggplot2” and “corrplot” were used for statistical analysis and visualization of the data [63]. Other statistical analyses were conducted using the GraphPad Prism 6 software. Data are represented as mean ± SD (standard deviation). Data obtained from in vitro assays were subjected to unpaired two-tailed t-test or one-way ANOVA with Tukey’s test for multiple comparisons. *P* < 0.05 was considered to indicate a statistically significant difference.

### Supplementary information

aj-checklist

Ethics approval and consent

supplemental figure1

supplemental figure2

supplemental figure3

supplemental figure4

supplementary figure legends

Supplementary Table1

Supplementary Table2

Supplementary Table3

## Data Availability

All data generated or analyzed during this study are included in this published article (and its supplementary information files).
