# Supplementary material for: RACK1 facilitates breast cancer progression by competitively inhibiting the binding of β-catenin to PSMD2 and enhancing the stability of β-catenin
Source: Cell Death Dis. 2023 Oct 17;14(10):685. doi: 10.1038/s41419-023-06191-3 (PMC10582012; doi:10.1038/s41419-023-06191-3)
Supplement: Supplementary file 2 — Ethics approval and consent [file 41419_2023_6191_MOESM2_ESM.pdf]

天津医科大学肿瘤医院实验动物伦理审批书

The Certification of the approval of The Laboratory Animal Ethic Committee

审批编号 Approved No.: AE-2021094

以下课题项目涉及动物实验经过实验动物伦理委员会审核，符合动物保护、动物福利和伦理原则，符合国家实验动物福利伦理的相关规定，特此证明。

The animal experiments involved in the following project has been reviewed and approved by the Animal Ethical and Welfare Committee (AEWC), Hereby certify。

科室<br>Name of department	公共实验室<br>Public laboratory	课题负责人<br>Principal investigator (PI)	牛瑞芳<br>Niu Ruifang
实验名称<br>Name of experiment	Rack1 通过抑制β-catenin 的降解增强 WNT 信号通路的分子机制研究<br>Molecular mechanism of Rack1 to enhance WNT signaling pathway by inhibiting degradation of β-catenin		
项目类别<br>Category of Project	国家自然科学基金<br>National nature science foundation	申请日期<br>Application Date	2020-10-20<br>10,20,2020
动物种系<br>Species or Strains	BALB/c nude	动物数量<br>Quantity	32（只）
实验动物来源<br>Source of laboratory animal	☑采购（purchase）<br>供应商（company）: 江苏集萃药康生物科技有限公司	生产许可证编号<br>Number of permit	SCXK（苏）2018-0008
	☐实验室自繁（reproduction）<br>实验室所属单位（lab）	设施使用许可证号<br>License number	
	☐赠与（donate）<br>赠与单位和赠与人（donor）	设施使用许可证号<br>License number	
饲养设施条件<br>Condition of the housing facilities	☑屏障设施 Barrier housing facility<br>☐普通设施 Ordinary housing facility		
许可证编号<br>Number of permit	SYXK（津）2017-0005	有效期：2017 年 9 月 8 日至 2022 年 9 月 7 日<br>Term of validity: Sept.8,2017- Sept.7,2022	
许可证发放机构<br>Permitting organization	天津市科学技术委员会<br>Tianjin Municipal Science and Technology Commission		
设施负责人意见<br>Chief Facility Officer	☑同意 Agree<br>☐不同意 Disagree		

审查意见（Result of inspection）：

符合动物伦理要求，可以进行实验（Agree）。

天津医科大学肿瘤医院实验动物伦理委员会（签章）
(Stamp of The Animal Ethical and Welfare Committee of Tianjin Medical University Cancer Institute and Hospital)

日期：2021 年 10 月 29 日
Date: 2021. 10. 29

备注 Supplement:
