# Supplementary figures and images for: RACK1 facilitates breast cancer progression by competitively inhibiting the binding of β-catenin to PSMD2 and enhancing the stability of β-catenin
Source: Cell Death Dis. 2023 Oct 17;14(10):685. doi: 10.1038/s41419-023-06191-3 (PMC10582012; doi:10.1038/s41419-023-06191-3)

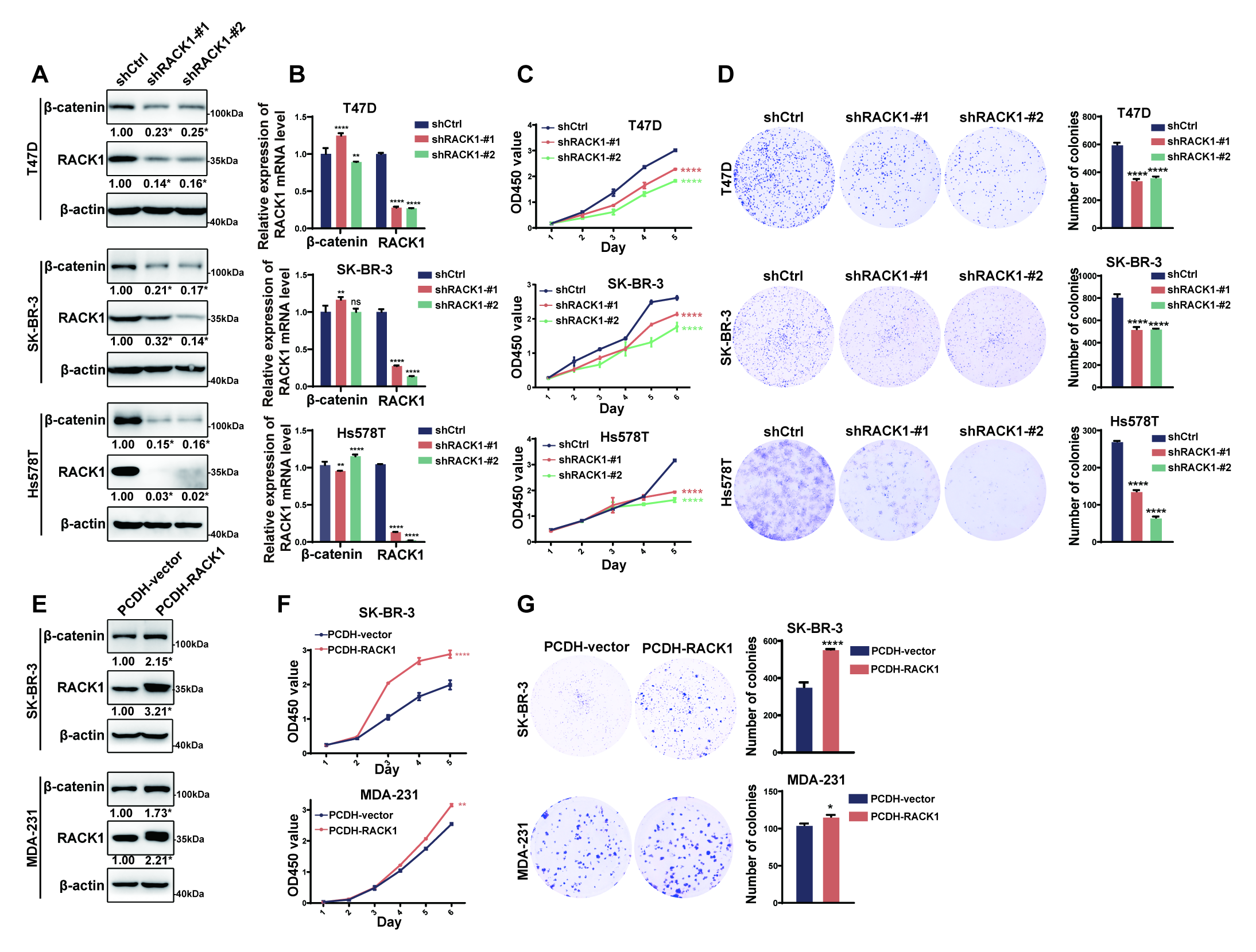

Supplement: Supplementary file 3 — supplemental figure1 [file 41419_2023_6191_MOESM3_ESM.tif]

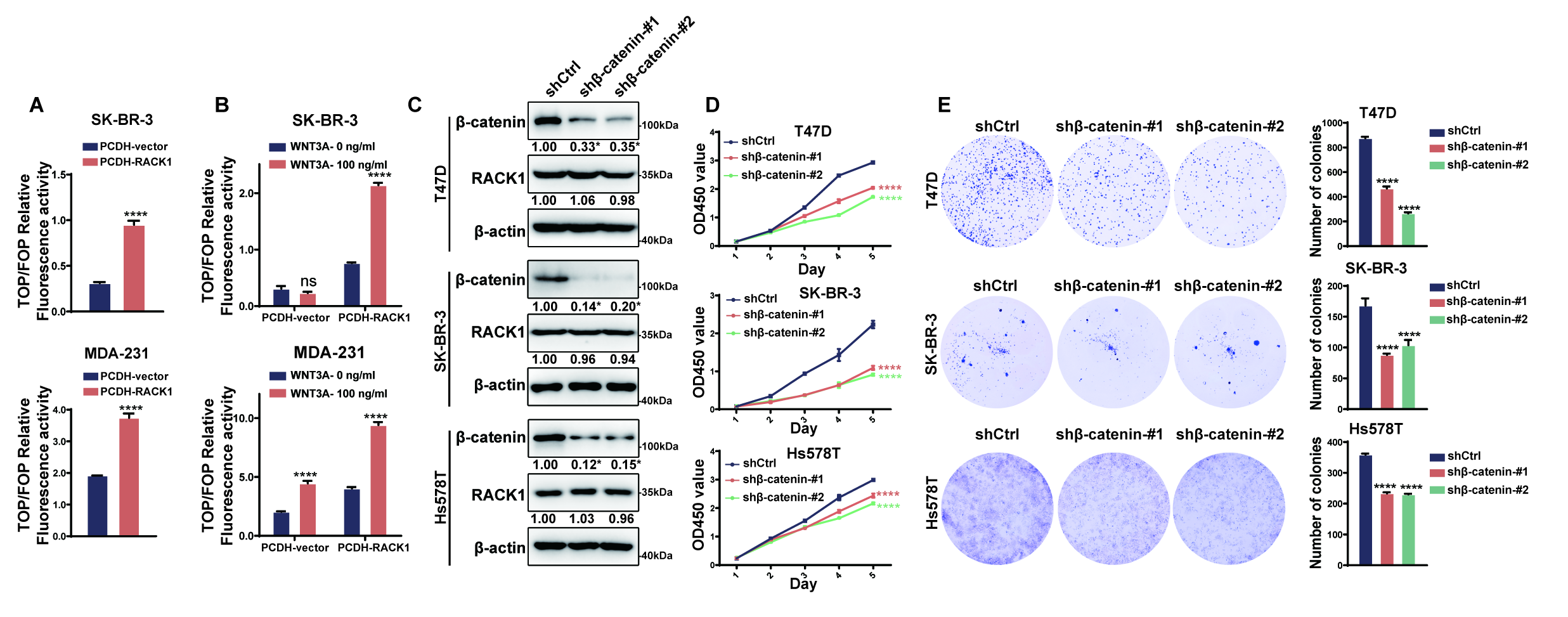

Supplement: Supplementary file 4 — supplemental figure2 [file 41419_2023_6191_MOESM4_ESM.tif]

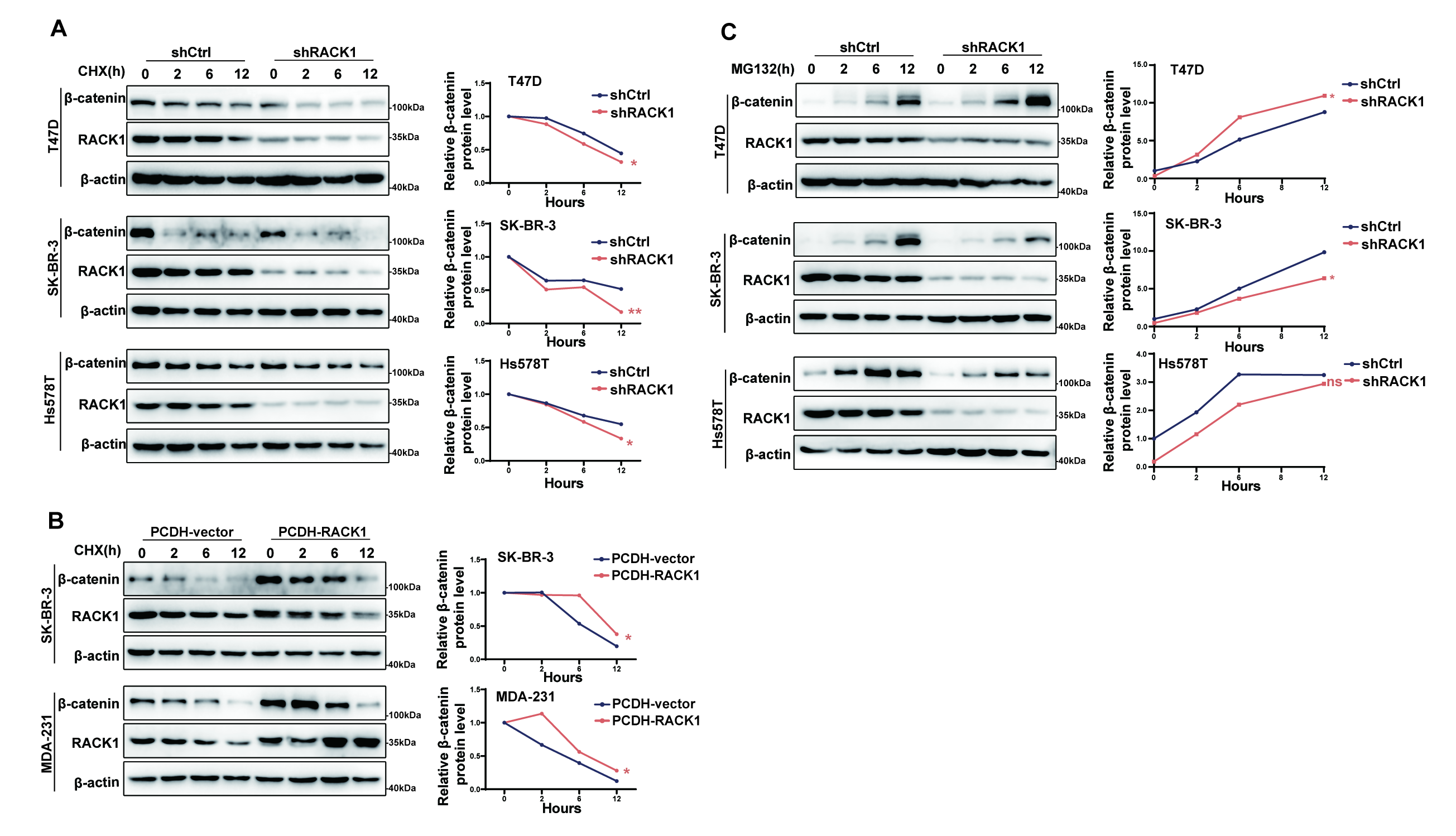

Supplement: Supplementary file 5 — supplemental figure3 [file 41419_2023_6191_MOESM5_ESM.tif]

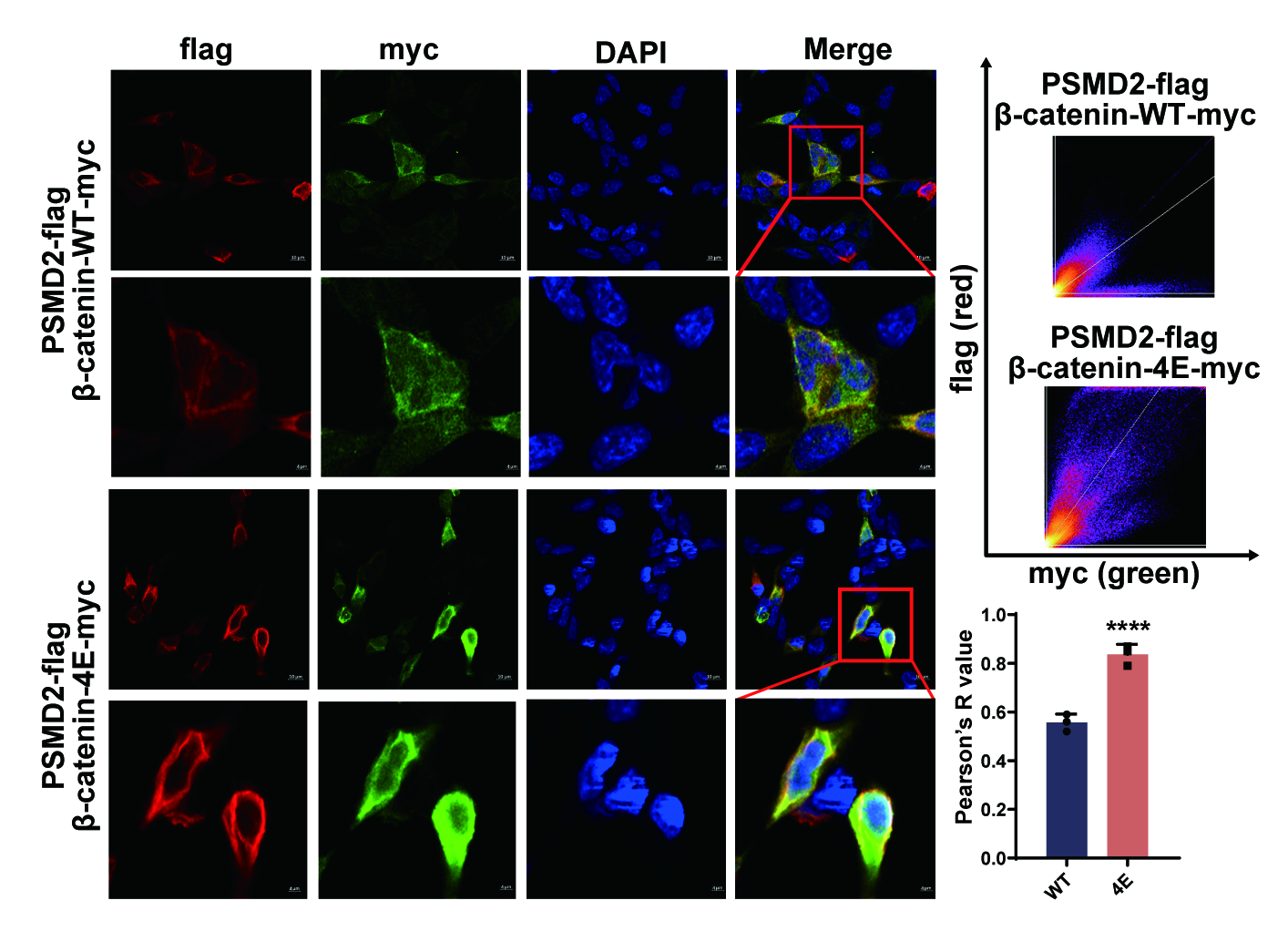

Supplement: Supplementary file 6 — supplemental figure4 [file 41419_2023_6191_MOESM6_ESM.tif]
