# Supplementary material for: RACK1 facilitates breast cancer progression by competitively inhibiting the binding of β-catenin to PSMD2 and enhancing the stability of β-catenin
Source: Cell Death Dis. 2023 Oct 17;14(10):685. doi: 10.1038/s41419-023-06191-3 (PMC10582012; doi:10.1038/s41419-023-06191-3)
Supplement: Supplementary file 7 — supplementary figure legends [file 41419_2023_6191_MOESM7_ESM.docx]

**Supplementary Fig 1. RACK1 is required for breast cancer cell proliferation. A**-**B** Western blotting (A) and qRT-PCR (B) analysis of RACK1 and β-catenin expression RACK1-silenced T47D, SK-BR-3 and Hs578T cells. **C** CCK8 analysis of the effect of RACK1 knockdown on the proliferative capacity of three breast cancer cell lines (two-way ANOVA test). **D** Colony formation assay showed that RACK1 knockdown decreased the proliferation rate of breast cancer cells. **E** Western blotting analysis of RACK1 and β-catenin expression in RACK1- elevated SK-BR-3 and MDA-MB-231 cells. **F** CCK8 analysis of the effect of RACK1 elevation on the proliferative capacity of SK-BR-3 and MDA-MB-231 cells (two-way ANOVA test). **G** Colony formation assay showed that RACK1 elevation promoted proliferation in SK-BR-3 and MDA-MB-231cells. The relative expression of the proteins was quantified by grayscale using Image J software. All data are expressed as the mean ± SD; *p < 0.05. **p < 0.01, ***p < 0.001, ****p < 0.0001 and ns p > 0.05 versus control, N = 3.

**Supplementary Fig 2. Silencing of β-catenin decreased the proliferative capacity of breast cancer cells. A-B** TOP Flash assays showed that basal (E) and Wnt3A-induced (F) β-catenin-dependent TCF/LEF transcriptional activity was significantly increased in RACK-overexpressed cells compared with control cells. **C** Western blotting analysis showed that silencing of β-catenin did not affect RACK1 expression in breast cancer cells. **D-E** Knockdown of β-catenin inhibited the proliferative capacity of breast cancer cells as measured by CCK-8 (B) and colony formation assay (C).The relative expression of the proteins was quantified by grayscale using Image J software. All data are expressed as the mean ± SD; *p < 0.05. **p < 0.01, ***p < 0.001, ****p < 0.0001 and ns p > 0.05 versus control, N = 3.

**Supplementary Fig 3. RACK1 regulates β-catenin stability through the ubiquitin-proteasome system.**

**A** Western blotting analysis of β-catenin expression in control and RACK1-silenced breast cancer cells treated with cycloheximide (CHX). The relative levels of β-catenin were quantified and are shown in the right panel. **B** Western blotting analysis of β-catenin expression in control and RACK1-elevated breast cancer cells treated with cycloheximide (CHX). The relative levels of β-catenin were quantified and are shown in the right panel. Gray scale analysis was performed using Image J software and the gray scale values of each band were normalized to the mean value of each group at 0 hour. **C** Western blotting analysis of β-catenin expression in control and RACK1-silenced breast cancer cells treated with 10 μM of MG132.The relative expression values of the proteins were normalized to those of the control at 0 hours.

**Supplementary Fig 4. β-catenin-4E co-localized more efficiently with PSMD2 than β-catenin-WT with PSMD2.** Immunofluorescence analysis of PSMD2-flag and β-catenin-WT-myc or β-catenin-4E-myc in HEK-293T cells.
